# Urinary Corticoid-to-Creatinine Ratio 8 Hours After Low-Dose Oral Dexamethasone for the Diagnosis of Cushing’s Syndrome in Dogs

**DOI:** 10.3390/ani16010084

**Published:** 2025-12-28

**Authors:** Elber Alberto Soler Arias, Hans S. Kooistra

**Affiliations:** 1 Unidad de Endocrinología, Hospital Escuela de Veterinaria, Universidad de Buenos Aires, Ciudad Autónoma de Buenos Aires, Av. San Martín 4351, Buenos Aires C1427CWO, Argentina; 2 Endovett, Servicio de Endocrinología Veterinaria, Ciudad Autónoma de Buenos Aires, Buenos Aires C1427CWO, Argentina; 3 Department of Clinical Sciences, Faculty of Veterinary Medicine, Utrecht University, Yalelaan 108, 3584 CM Utrecht, The Netherlands; h.s.kooistra@uu.nl

**Keywords:** hyperadrenocorticism, hypercortisolism, oral dexamethasone, at-home diagnostic test, urinary cortisol, dexamethasone suppression test

## Abstract

The intravenous low-dose dexamethasone suppression test (IV-LDDST) is considered the most accurate method for diagnosing Cushing’s syndrome (CS) in dogs, but its intravenous administration and repeated hospital sampling are stressful and time-consuming for both dogs and owners. This study evaluated an oral LDDST (O-LDDST) as a minimally invasive alternative. The approach is based on the urinary corticoid-to-creatinine ratio (uCC) measured in urine samples collected at home at baseline (B-uCC) and 8 h after administering a low oral dose of dexamethasone (0.01 mg/kg; 8h-uCC). A total of 168 client-owned dogs were prospectively recruited: 42 healthy dogs (HD), 40 dogs with disease mimicking CS (DMCS), and 86 dogs with confirmed CS. An ROC curve analysis showed that dogs with CS had significantly higher B-uCC and 8h-uCC and a lower suppression percentage (%S) compared with HD and DMCS (all *p* < 0.0001). Optimal cut-offs were B-uCC > 14.2 × 10^−6^, 8h-uCC > 6.7 × 10^−6^, and %S < 48.6%, yielding sensitivity/specificity of 82.9%/83.3%, 95.3%/92.6%, and 86.0%/85.3%, respectively. O-LDDST was preferred by 85.9% of owners, although it did not distinguish pituitary- from adrenal-dependent CS. The O-LDDST proved to be a reliable, accurate, and minimally invasive diagnostic option for CS in dogs, with 8h-uCC providing the highest accuracy.

## Simple Summary

The lack of suppression of serum cortisol 8 h after a low dose of intravenous dexamethasone is the most accurate test for diagnosing Cushing’s syndrome in dogs, but its performance may cause stress for both the dog and owner because it requires several venipunctures. To reduce this problem, the test was evaluated in a home setting by administering a low dose of dexamethasone orally and measuring the urinary corticoid-to-creatinine ratio before and 8 h after administration. In 168 dogs (86 with Cushing’s syndrome), the 8 h sample showed high diagnostic accuracy for Cushing’s syndrome, with a sensitivity of 95% and specificity of 93%. In addition, its preference by 86% of owners over the intravenous version supports the oral method as a valid and clinically useful diagnostic option.

## 1. Introduction

Spontaneous Cushing’s syndrome (CS), or hypercortisolism, is a clinical syndrome caused by chronic excessive endogenous glucocorticoid activity [1]. Although CS is among the most common endocrine disorders in dogs, its diagnosis still requires the integration of clinical symptoms, physical examination findings, hematological and serum biochemical abnormalities, and specific hormonal tests, as no single test provides 100% sensitivity and specificity [2].

Hormonal confirmation of CS relies on demonstrating cortisol excess due to impaired sensitivity of the hypothalamic–pituitary–adrenal axis to glucocorticoid negative feedback [2]. In healthy dogs, glucocorticoids suppress adrenocorticotropic hormone (ACTH) and cortisol secretion, whereas this feedback is blunted in dogs with CS. Consequently, the low-dose dexamethasone suppression test (LDDST) is considered the diagnostic test of choice, with reported sensitivity of 85–100% and specificity of 67–80% [3,4]. However, the standard intravenous LDDST (IV-LDDST) requires three clinic visits and three to four venipunctures (baseline sampling, dexamethasone administration, and two post-injection samples), which is time-consuming and stressful for both dogs and owners.

The urinary corticoid-to-creatinine ratio (uCC) can also be used to demonstrate cortisol excess, but it does not assess the sensitivity of the feedback system. Nevertheless, it can aid in diagnosing CS when appropriate cut-off values and assay methods are applied [5]. Its specificity is limited, particularly with chemiluminescent immunoassays (CLIA), in dogs with diseases mimicking CS (DMCS), in those receiving glucocorticoids, or when urine is collected under stressful conditions such as hospitalization [6,7,8,9]. A major advantage of the uCC is that a urine sample can be collected at home, in a stress-free environment [10].

Another dynamic diagnostic test for CS is the ACTH stimulation test (ACTHst), which shows lower sensitivity and specificity than the IV-LDDST [9]. As with the IV-LDDST, it requires a hospital stay of at least 2 h, two to three venipunctures, and the use of synthetic ACTH, which is not available in many countries.

Based on previous findings showing that the same low dose of dexamethasone (0.01 mg/kg) used in the IV-LDDST, when administered orally, suppressed uCC at 8 h (8h-uCC) in healthy dogs, we aimed to determine whether a LDDST performed at home, using oral dexamethasone (O-LDDST) and measuring baseline (B-uCC) and 8h-uCC, could serve as a diagnostic tool for CS in dogs, and to evaluate its diagnostic accuracy and practical applicability [11]. In addition, we sought to assess owners’ preferences regarding O-LDDST versus IV-LDDST.

## 2. Materials and Methods

### 2.1. Animals

This was a prospective diagnostic accuracy study that included client-owned dogs recruited between October 2022 and April 2025 at an endocrinology referral center in Buenos Aires, Argentina.

Inclusion criteria for CS required the presence of clinical signs (e.g., polyuria, polydipsia, polyphagia, and panting), physical examination findings (e.g., non-pruritic alopecia, pot-bellied appearance, and thin skin), laboratory abnormalities (e.g., elevated alkaline phosphatase activity, increased fasting total cholesterol, and low urinary specific gravity), and a positive result on a dynamic test, either the IV-LDDST or ACTHst, or both [6]. Adrenal-dependent CS (A-CS) was diagnosed when the plasma endogenous ACTH concentration was undetectable (<10 pg/mL), and was associated with either a unilateral adrenal tumor and an atrophied contralateral adrenal gland [12], or with bilateral adrenal tumors. Pituitary-dependent CS (P-CS) was diagnosed by the absence of an adrenal tumor on ultrasonography, in association with pituitary changes on computed tomography (CT, including the pituitary height-to-brain area ratio) consistent with a pituitary tumor [13] or with the endogenous ACTH concentration > 10 pg/mL (Supplementary Material S1).

Dogs were excluded from the study if they failed to meet inclusion criteria, had a severe systemic illness (e.g., renal or hepatic failure, inflammatory bowel disease, or protein-losing enteropathy), or had received recent treatment (<6 weeks) with trilostane, mitotane, ketoconazole, butorphanol, glucocorticoids, or progestins.

A group of dogs with diseases mimicking CS (DMCS) was included based on clinicopathological findings compatible with CS, but with negative results on endocrine dynamic tests (IV-LDDST or ACTHst), and with confirmation of an alternative diagnosis that accounted for the clinical signs, followed by their remission in cases where specific treatment was possible. Dogs with a histopathological diagnosis of pheochromocytoma or non-secreting adrenocortical tumors were also included in the DMCS group.

Additionally, a control group of clinically healthy dogs (HD) was selected based on the absence of clinical signs, unremarkable findings on physical examinations, and normal results on routine laboratory testing (hematology, serum biochemistry, and urinalysis), as well as on abdominal ultrasonography.

### 2.2. Collection and Processing of Urine Samples

Owners received written instructions for performing the O-LDDST, along with a customized dose of dexamethasone sodium phosphate reformulated into 0.1 mg and 0.2 mg tablets using lactose and microcrystalline cellulose as excipients. A tablet combination was provided according to the calculated dose (0.01 mg/kg) for each dog.

Urine collection was performed at home by owners, at least five days after the consultation visit [11]. Two urine samples were collected in separate silicone containers: the first at 8:00 a.m. (first morning urine) and the second at 4:00 p.m. (8 h later). Immediately after the first sample, dexamethasone was administered orally, mixed with a small amount of food. At 12:00 p.m. (4 h post-administration), owners were instructed to walk their dog to promote bladder emptying. Both samples were refrigerated, transported to the laboratory the following day [8]. Urine was collected either directly during midstream urination or indirectly from a clean surface and then transferred to a sealed container.

### 2.3. Hormonal Determinations

Urinary cortisol concentration was measured using a chemiluminescent microparticle immunoassay (CMIA; kit 8D15-25) on an Architect i2000sr analyser (Abbott Laboratories, Abbott Park, IL, USA). As no previous validation studies were available for veterinary use, a functional validation protocol was conducted by comparing the results with those obtained using the Immulite 1000 cortisol assay validated in dogs (Supplementary Material S2). The lower limit of detection of the assay for cortisol was 22 nmol/L (0.8 µg/dL). Urinary creatinine concentration was determined using an automated kinetic Jaffe method (Wiener Laboratory, Rosario, Argentina). Cortisol concentration (nmol/L) was correlated to urinary creatinine concentration (µmol/L) and expressed as a ratio (uCC × 10^−6^), following previously reported protocols [6,14].

Serum cortisol for the IV-LDDST and ACTHst, as well as endogenous ACTH (eACTH) concentrations, were determined by chemiluminescence immunoassay (CLIA, Immulite 1000, Siemens Healthineers, Tarrytown, NY, USA) validated for dogs [15,16,17]. The lower limits of quantification for cortisol and eACTH were 27.5 nmol/L (1 µg/dL) and 10 pg/mL, respectively.

Blood samples for cortisol were collected in serum-separating tubes, allowed to clot, centrifuged for 10 min at 3000× *g*, immediately transferred to plastic tubes, stored at −20 °C, and analyzed within 24 h. For ACTHst, blood samples were collected before and 60 and 120 min after intramuscular injection of 2.2 U/kg synthetic ACTH (Corticotrophin, ACTHEL; GP Pharma) [18]. Samples for eACTH determination were collected on the same day as the LDDST, prior to dexamethasone injection, using EDTA-coated plastic tubes placed on ice. Plasma was separated by centrifugation at 1000× *g* for 10 min, transferred to plastic tubes, stored at −20 °C, and analyzed within 24 h [19].

All tests were performed in a private institution, and analytical procedures were carried out at the veterinary laboratory of the Hospital School of the University of Buenos Aires.

### 2.4. Owner Preference

Owner preference was assessed only in cases with confirmed CS, using a single direct question at the end of the study: “Which test would you prefer for your dog: O-LDDST or IV-LDDST?” Responses were recorded as categorical variables (O-LDDST or IV-LDDST).

### 2.5. Statistical Analysis

Statistical analysis was performed using GraphPad Prism 10 (GraphPad software, San Diego, CA, USA). Data distribution was assessed with the Kolmogorov–Smirnov test. Results are expressed as ranges, medians, and interquartile ranges (IQR). Group comparisons were performed using the non-parametric Kruskal–Wallis test, and effect size estimates (median differences) with 95% confidence intervals (95% CI) were calculated and reported together with *p*-values. A receiver operating characteristic (ROC) curve was generated to determine the optimal cut-off value for B-uCC, 8h-uCC, and %S, and to calculate sensitivity, specificity, and area under the curve (AUC) with corresponding 95% CI. A *p*-value < 0.05 was considered statistically significant.

## 3. Results

### 3.1. Animals

A total of 168 dogs were enrolled and divided into three groups: 86 dogs with confirmed CS (group CS), 40 dogs with diseases mimicking CS (group DMCS), and 42 clinically healthy dogs (group HD, controls).

Eighty-six dogs with CS were included: 67 with pituitary-dependent CS and 19 with adrenal-dependent CS (Supplementary Material S1): fifty-five females (45 spayed) and 31 males (25 neutered). The median age was 7.0 years (range, 2–14 years), and the median body weight was 8.3 kg (range, 1.3–45 kg). Twenty-two dogs were mongrels, while the remaining 64 represented 19 different breeds: 27 Poodles, 5 Boxers, 4 Yorkshire Terriers, 3 Pit Bulls, 3 Schnauzers, 3 Miniature Pinscher, 2 Dachshunds, 2 Golden Retrievers, 2 Bichon Frises, 2 Shih Tzus, 2 Beagles, 2 Maltese, and one of each of Labrador Retriever, Jack Russell Terrier, Fox Terrier, English Bulldog, French Bulldog, Shetland Sheepdog, and White Swiss Shepherd Dog.

Forty dogs with DMCS were included: 22 males (18 neutered) and 18 females (17 spayed). The median age was 9.0 years (range, 2–16 years), and the median body weight was 9.7 kg (range, 1.6–44.6 kg). Seven dogs were mongrels, and the remaining 33 represented 12 different breeds: 11 Poodles, 6 Schnauzers, 4 Yorkshire Terriers, 3 Dachshunds, 2 English Cocker Spaniels, and one each of Labrador Retriever, Bichon Frise, Pit Bull Terrier, Beagle, Miniature Pinscher, Maltese, and Shih Tzu. Final diagnoses in this group were pheocromocytoma (*n* = 5), non-secreting adrenal tumors (*n* = 4), hypothyroidism (*n* = 3), alopecia X (*n* = 3), sudden acquired retinal degeneration syndrome (SARDs; *n* = 3), diabetes mellitus (*n* = 5), primary hyperparathyroidism (*n* = 2), brain neoplasia (*n* = 3), obesity-associated dyslipidemia (*n* = 6), and chronic hepatopathy (*n* = 6).

Forty-two HD were included: 23 females (22 spayed) and 19 males (18 neutered). The median age was 9.2 years (range, 2–15 years), and the median body weight was 14.7 kg (range, 2.8–46.5 kg). Eleven dogs were mongrels, while the remaining 31 represented 16 breeds: 4 Poodles, 4 Jack Russells Terriers, 3 Golden Retrievers, 3 Labrador Retrievers, 3 Beagles, 2 Yorkshire Terriers, 2 Dachshunds, 2 West Highland White Terriers, and one each of French Bulldog, Boston Terrier, Old English Sheepdog, Border Collie, Bichon Frise, Pit Bull, Bernes Mountain dog, and Schnauzer.

### 3.2. Basal Urinary Corticoid-to-Creatinine Ratio (B-uCC)

**Figure 1:**
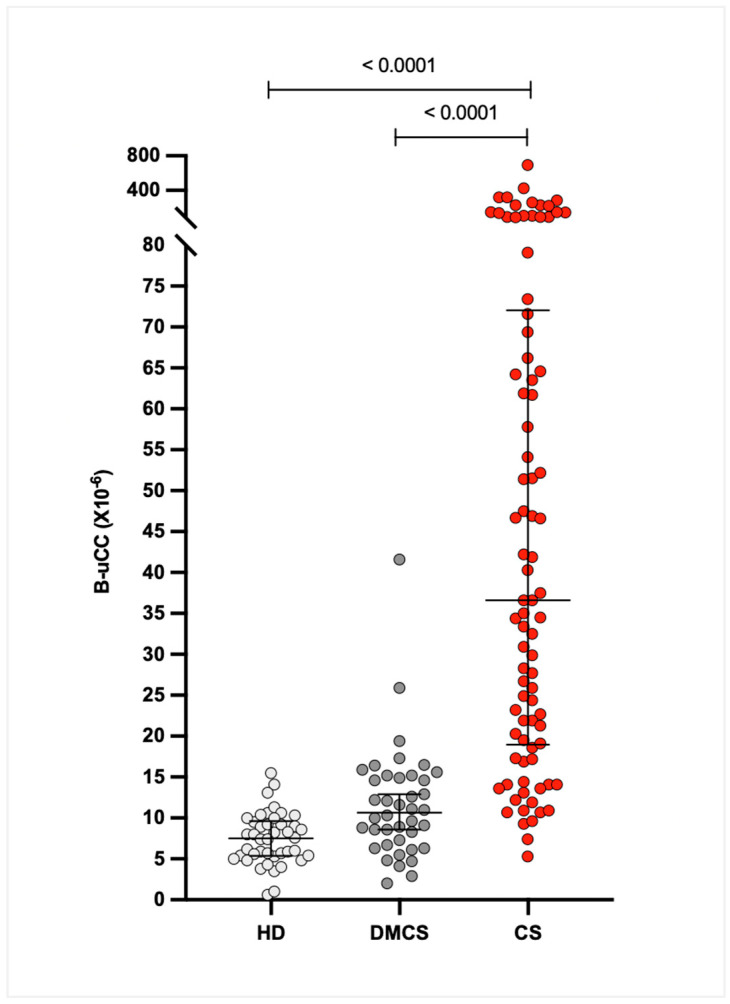
Comparison of baseline urinary corticoid-to-creatinine ratio (B-uCC, ×10^−6^) among healthy dogs (HD, n = 42), dogs with diseases mimicking Cushing’s syndrome (DMCS, n = 40), and dogs with Cushing’s syndrome (CS, n = 86). Each dot represents an individual dog. Horizontal bars indicate medians with interquartile ranges.

The median (IQR) B-uCC values were 36.6 × 10^−6^ (21.6–70.8 × 10^−6^) for dogs with CS, 10.9 × 10^−6^ (6.6–14.8 × 10^−6^) for dogs with DMCS, and 7.4 × 10^−6^ (5.3–9.8 × 10^−6^) for HD. The B-uCC ratio was significantly higher in dogs with CS compared to DMCS (median difference 26.0 × 10^−6^, 95% CI 17.3–38.9 × 10^−6^; *p* < 0.0001) and HD (median difference 29.1 × 10^−6^, 95% CI 20.7–41.6 × 10^−6^; *p* < 0.0001). No significant difference was observed between DMCS and HD (median difference 3.2 × 10^−6^, 95% CI 0.7–6.3 × 10^−6^; *p* = 0.07; Figure 1).

### 3.3. Eight-Hour Urinary Cortisol-to-Creatinine Ratio (8h-uCC)

**Figure 2:**
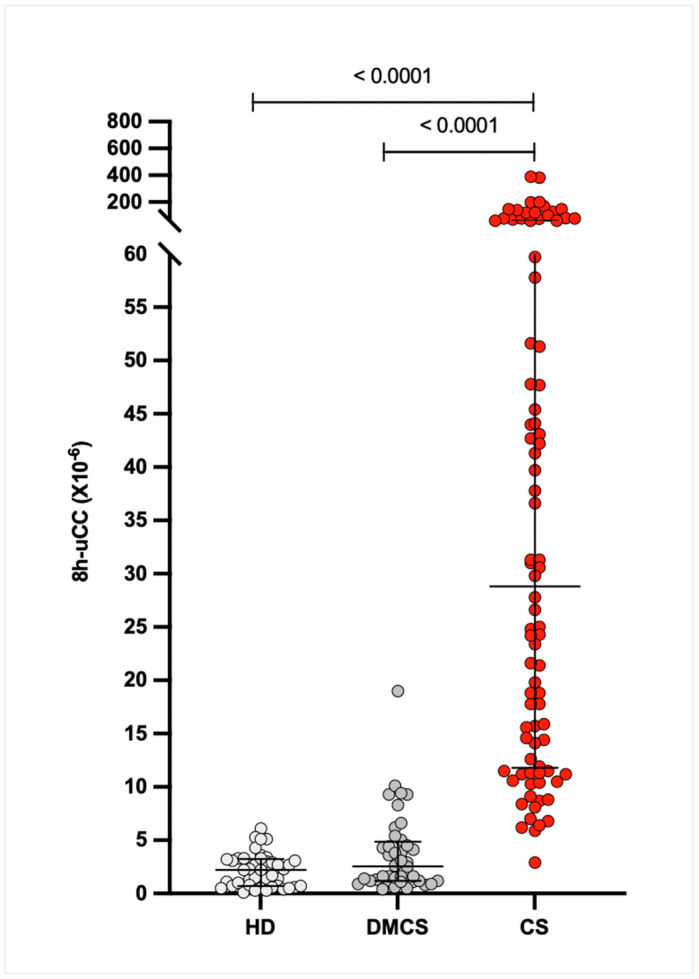
Comparison of urinary corticoid-to-creatinine ratio 8 h after oral low-dose dexamethasone (8h-uCC, ×10^−6^) among healthy dogs (HD, n = 42), dogs with diseases mimicking Cushing’s syndrome (DMCS, n = 40), and dogs with Cushing’s syndrome (CS, n = 86). Each dot represents an individual dog. Horizontal bars indicate medians with interquartile ranges.

The median (IQR) 8h-uCC values were 28.8 × 10^−6^ (12.0–60.3 × 10^−6^) for dogs with CS, 2.5 × 10^−6^ (1.2–4.6 × 10^−6^) for dogs with DMCS, and 2.2 × 10^−6^ (0.7–3.1 × 10^−6^) for HD. The 95% CIs for the medians were 18.6–47.8 × 10^−6^ for CS, 1.4–3.8 × 10^−6^ for DMCS, and 1.4–3.3 × 10^−6^ for HD. The 8h-uCC ratio was significantly higher in dogs with CS compared with DMCS and HD (*p* < 0.0001), with no significant difference between DMCS and HD (*p* = *0*.07; Figure 2).

### 3.4. Percentage Suppression (%S)

**Figure 3:**
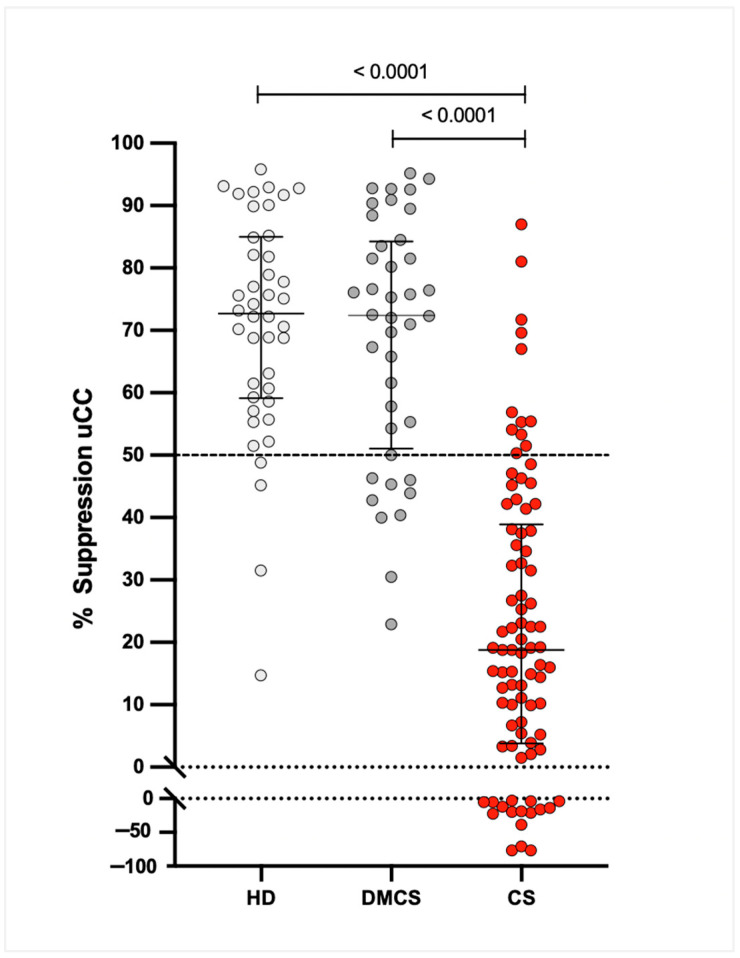
Percentage suppression (%S) of the urinary corticoid-to-creatinine ratio (uCC) in healthy dogs (HD, n = 42), dogs with diseases mimicking Cushing’s syndrome (DMCS, n = 40), and dogs with confirmed Cushing’s syndrome (CS, n = 86), measured 8 h after oral administration of low-dose dexamethasone. Each dot represents an individual dog. Horizontal bars indicate medians with interquartile ranges. The lower dotted line indicates negative suppression values.

Median (IQR) %S values were 18.8% (4.2–38.0%) for dogs with CS, 72.4% (53.2–83.7%) for dogs with DMCS, and 72.7% (59.6–84.2%) for HD. The 95% CIs for the medians were 11.1–28.3% for CS, 63.5–79.8% for DMCS, and 64.3–78.9% for HD. The %S was significantly lower in dogs with CS compared to both DMCS and HD (*p* < 0.0001), with no significant difference between DMCS and HD (Figure 3).

### 3.5. Sensitivity and Specificity: B-uCC, 8h-uCC, and %S

**Figure 4:**
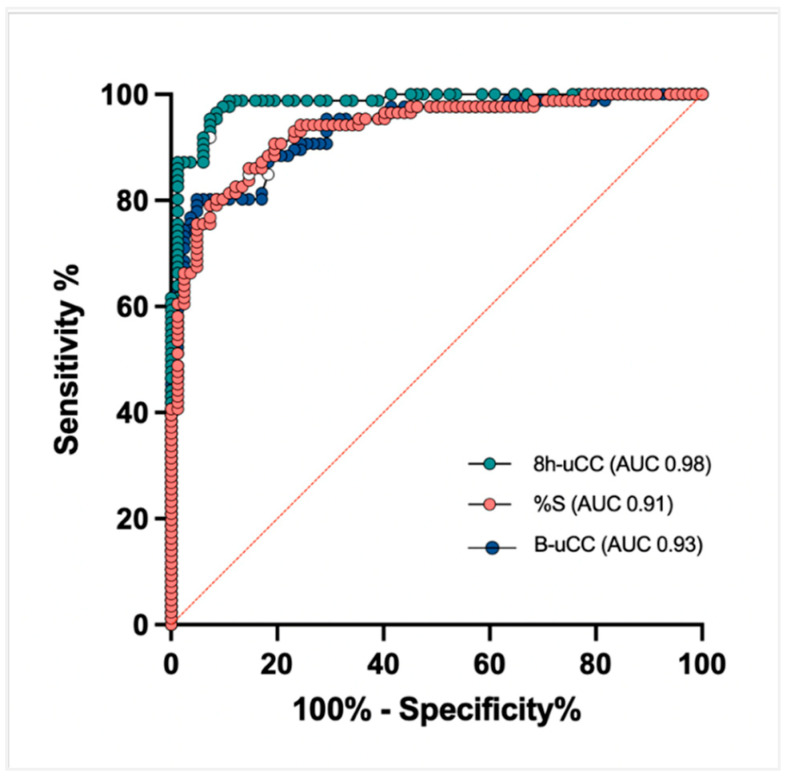
Receiver operating characteristic (ROC) curves for B-uCC, 8h-uCC, and %S to differentiate dogs with Cushing’s syndrome (CS) from dogs with disease mimicking CS (DMCS) and healthy dogs (HD).

A receiver operating characteristic (ROC) analysis identified a B-uCC (>14.2 × 10^−6^), 8h-uCC (>6.7 × 10^−6^), and %S < 48.6% as the optimal cut-off for CS diagnosis. Using this cut-off, the sensitivity for diagnosing CS was 81.4%, 95.3%, and 86.0%, respectively, while specificity was 82.9%, 92.6% and 85.3%, respectively. According to the area under the curve ROC curve, 8h-uCC (0.98 [95% CI, 96–99%]) had the greatest discriminatory capacity for diagnosing CS, compared to B-uCC (0.93 [95% CI, 89–96%]) and %S (0.91 [95% CI, 89–96%]) (Figure 4).

A sensitivity and specificity of 100% for B-uCC, 8h-uCC, and %S could be achieved by lowering or increasing the established cut-off values, respectively (Table 1).

**Figure 5:**
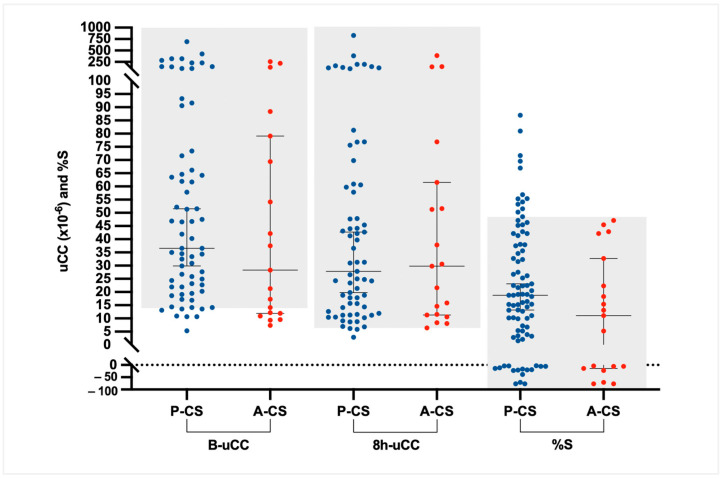
Comparison of baseline urinary corticoid-to-creatinine ratio (B-uCC), urinary corticoid-to-creatinine 8 h after oral low-dose dexamethasone administration (8h-uCC), and percent suppression (%S) between dogs with pituitary-dependent Cushing’s syndrome (P-CS, n = 67) and adrenal-dependent Cushing’s syndrome (A-CS, n = 19). Each dot represents an individual dog. Horizontal bars indicate medians and interquartile ranges. Gray-shaded areas indicate values within the diagnostic cut-off ranges established in this study. The dotted line on the *Y*-axis denotes 0% suppression.

In dogs with CS, B-uCC, 8h-uCC, and %S values did not differ significantly between dogs with P-CS (median [IQR]: 36.6 [21.9–68.9] × 10^−6^; 27.8 [12.0–74.2] × 10^−6^; and 18% [4.2–38.0%]) and those with A-CS (median [IQR]: 28.3 [12.0–74.2] × 10^−6^; 29.8 [11.4–56.5] × 10^−6^; and 11.1% [−9.6–27.5%]). However, all dogs with A-CS (19/19; 100%) had %S < 50% (Figure 5); 74 of the 86 dogs with CS (86%) showed a %S below 50% at 8 h after dexamethasone administration.

### 3.6. Owner Preference

Of the 86 dogs with CS, IV-LDDST was performed in 57 (38 with pituitary-CS and 19 with adrenal-CS). Overall, 49 owners (86%) preferred O-LDDST. Eight owners chose IV-LDDST; based on their spontaneous comments, three reported losing the dexamethasone dose provided and therefore had to return to the clinic for a replacement, four reported difficulties collecting urine at the scheduled times, and one considered blood sampling more convenient than urine collection.

## 4. Discussion

This prospective study demonstrates that an at-home O-LDDST, evaluated through uCC, can accurately discriminate among dogs with spontaneous CS from both HD and those with DMCS. Among the parameters tested, the 8h-uCC achieved the highest diagnostic accuracy, with sensitivity and specificity exceeding 90%, and an area under the ROC curve of 0.98. These results support the use of the O-LDDST as a reliable and minimally invasive diagnostic test for CS in dogs.

The IV-LDDST remains the most widely recommended screening test for canine CS, with a reported sensitivity between 85 and 100% and a specificity of 67–80% [2,3,4]. However, performing the IV-LDDST requires multiple hospital visits and venipunctures, which may be time-consuming for the veterinarian and stressful for both dogs and owners. Stress associated with hospital visits and venipuncture is a well-recognized confounder in cortisol assessment. In healthy dogs, the serum cortisol concentration and heart rate were significantly higher after waiting in a veterinary clinic environment compared with waiting outdoors, highlighting the acute stress response elicited by the hospital visit [9]. Similarly, uCC ratios have been shown to be significantly higher in samples collected at the hospital compared with those obtained at home [20]. Together, these findings underscore the impact of the clinical environment on cortisol measurements and reinforce the rationale for O-LDDST, which minimizes stress-related artifacts and may enhance owner compliance.

Our findings suggest that the diagnostic performance of O-LDDST, particularly using the 8h-uCC cut-off, is comparable or even superior to the previously reported results for the IV-LDDST in terms of specificity, while maintaining similar sensitivity [21].

Basal uCC values were significantly higher in dogs with CS compared with HD and DMCS, but overlap between groups limits their stand-alone diagnostic value. This finding is consistent with previous reports indicating that uCC has low diagnostic specificity, as it can be influenced by stress, concurrent illness, or assay variability [6,7,8,9]. Although a B-uCC cut-off with 100% specificity (41.7 × 10^−6^ in our assay) may help identify CS in highly selected cases—provided samples are collected at home and in the absence of prior glucocorticoid or progestagen administration—this threshold would exclude more than half of affected dogs, limiting its clinical use. In our study, the sensitivity of B-uCC (81.4%) was comparable to that reported previously (~80%), whereas specificity was moderately higher (82.9% vs. ~70%), likely reflecting the referral nature of our population, where dogs are typically presented with a strong suspicion of endocrine disease [5,9,10].

In our cohort, the 8h-uCC provided the best diagnostic performance (AUC = 0.98), with sensitivity and specificity both >90%, comparing favourably with published IV-LDDST performance [2,3,4]. Biologically, the 8 h time point likely captures a robust dexamethasone effect while leveraging two advantages of the O-LDDST protocol: (a) at-home urine collection, which reduces stress-related cortisol elevations, and (b) the uCC metric, which integrates cortisol secretion over several hours and is therefore less vulnerable to single-sample pulsatility than serum cortisol. Despite its superior accuracy, the 8h-uCC did not differentiate pituitary- from adrenal-dependent CS, whereas IV-LDDST patterns at 4 h after dexamethasone can sometimes aid etiologic discrimination and help assess the reliability of a positive test result—albeit with recognized limitations and susceptibility to similar confounders. Overall, the data support 8h-uCC as the most informative O-LDDST endpoint for case identification, while acknowledging that etiologic classification still relies on complementary testing.

The %S also showed good discriminatory capacity and was comparable to the 50% suppression criterion used in the IV-LDDST, although it was less accurate than 8h-uCC. As in the IV-LDDST, variability in urinary basal cortisol or B-uCC may influence the calculated degree of suppression, thereby limiting its accuracy. In the IV-LDDST, partial suppression at 4 h may help differentiate between pituitary- and adrenal-dependent CS, an aspect not evaluated in the present study, in which the 8h-uCC did not allow discrimination of CS origin. However, no dog with adrenal-dependent CS showed a %S greater than 50%. It should be noted that assessing the utility of %S for etiologic discrimination was not among the primary objectives of this study [4,18].

In dogs, cortisol has been most extensively validated using a chemiluminescent immunoassay (CLIA), with analyzers such as Immulite 1000 or 2000 showing acceptable analytical performance [6,22]. In the present study, urinary cortisol was measured with a CMIA, and to support this approach, we provided a supplementary validation against CLIA, demonstrating good agreement between analyzers. Nevertheless, minor variability may affect absolute cortisol values and should be considered when comparing diagnostic cut-offs across studies [8,9]. The main clinical relevance of our work is that the O-LDDST can be reliably applied as a diagnostic tool for canine CS. However, diagnostic cut-offs must be determined individually for each analyzer, given the potential for variability [8].

An important strength of this study was the assessment of owner preferences: 85.9% favored O-LDDST over IV-LDDST, reflecting its greater practicality and comfort. A minority preferred IV-LDDST, suggesting that test choice may need to be individualized. Although stress was not objectively measured, the clear preference for O-LDDST supports the perception that it is less stressful for dogs and their owners.

This study has several limitations. Oral dexamethasone absorption may vary among dogs. In healthy animals, oral dosing reduced uCC by >50% at 8 h, though inter-individual variation was observed [11], and circulating dexamethasone concentrations have been shown to differ in dogs with CS compared with HD or non-adrenal diseased dogs [23]. Diagnostic accuracy was assessed against the IV-LDDST and the ACTHst. Although both are widely recommended, they do not constitute a perfect gold standard, and therefore, some degree of misclassification cannot be excluded. The study was conducted in a single referral endocrinology center, limiting external generalizability, and although the overall sample size was relatively large, subgroup analyses remain underpowered. Stress was not objectively measured, which might have strengthened the evaluation of owner-reported perceptions. Finally, cortisol was measured using a CMIA, not previously validated in dogs. However, a supplementary validation against CLIA confirmed acceptable agreement, and the diagnosis of CS was corroborated by additional methods performed with the validated Immulite 1000 analyzer. In addition, although CLIA may show greater inter-assay variability than a radioimmunoassay for cortisol measurement, limitations related to availability and radiation safety hazards for personnel restrict the routine use of radioimmunoassay [6,24]. Finally, further comparative studies evaluating the O-LDDST and IV-LDDST within the same patient population are warranted.

## 5. Conclusions

Taken together, our findings indicate that the O-LDDST is a practical, minimally invasive, and accurate alternative to the IV-LDDST for the diagnosis of CS in dogs. By combining oral dexamethasone administration with home urine collection, this approach may reduce patient stress, enhance owner compliance, and preserve high diagnostic accuracy, with the 8h-uCC showing the best performance among the evaluated parameters. However, diagnostic cut-off values for the O-LDDST must be established individually for each analyzer, given the variability associated with CLIA methods.

## Figures and Tables

**Table 1:** Sensitivity, specificity, positive predictive value (PPV), and negative predictive value (NPV) for different cut-off values of B-uCC, 8h-uCC, and %S in diagnosing Cushing’s Syndrome in dogs.

Parameter	Cut-Off	% Sensitivity (95% IC)	% Specificity (95% IC)	PPV (%)	NPV (%)
B-uCC (×10^−6^)	>5.1	100 (95.7–100%)	17.0 (10.4–26.4%)	55.8	100
	>14.2	81.4 (71.8–88.2%)	82.9 (73.3–89.5%)	83.3	80.9
	>41.7	46.5 (36.3–56.9%)	100 (95.2–100%)	100	64.1
8h-uCC (×10^−6^)	>9.3	100 (95.7–100%)	56.8 (47.1–66.0%)	70.6	100
	>6.7	95.3 (88.6–98.1%)	92.6 (84.9–96.6%)	93.7	94.6
	>19.4	61.6 (51.0–71.2%)	100 (95.2–100%)	100	72.2
%S	<87.7	100 (95.7–100%)	21.9 (14.3–32.0%)	56.5	100
	<48.6	86.0 (77.1–91.8%)	85.3 (76.1–91.4%)	85.7	85.6
	<14.5	40.7 (30.9–51.2%)	100 (95.5–100%)	100	61.7

## Data Availability

The data supporting the findings of this study are included within the article and its Supplementary Materials. Additional information is available from the corresponding author upon reasonable request.

## Supplementary Materials

The following supporting information can be downloaded at: https://www.mdpi.com/article/10.3390/ani16010084/s1, S1: Diagnostic criteria for dogs with CS; S2: Analytical validation of urinary cortisol.

## Abbreviations

The following abbreviations are used in this manuscript:

A-CS	adrenal-dependent Cushing’s syndrome
ACTH	adrenocorticotropic hormone
ACTHst	ACTH stimulation test
AUC	area under the curve
B-uCC	baseline urinary corticoid-to-creatinine ratio
CI	confidence interval
CLIA	chemiluminescent immunoassay
CMIA	chemiluminescent microparticle immunoassay
CS	Cushing’s syndrome
CT	computed tomography
DMCS	diseases mimicking Cushing’s syndrome
eACTH	endogenous adrenocorticotropic hormone
HD	healthy dogs
IQR	interquartile range
IV-LDDST	intravenous low-dose dexamethasone suppression test
LDDST	low-dose dexamethasone suppression test
O-LDDST	oral low-dose dexamethasone suppression test
P-CS	pituitary-dependent Cushing’s syndrome
ROC	receiver operating characteristic
S%	suppression percentage
uCC	urinary corticoid-to-creatinine ratio

## References

[1] Niessen S.J.M., Behrend E.N., Fracassi F., Church D.B., Foster S.F., Galac S., Melian C., Pöppl Á.G., Ramsey I.K., Sieber-Ruckstuhl N.S. (2025). Agreeing language in veterinary endocrinology (ALIVE): Cushing’s syndrome and hypoadrenocorticism-A modified Delphi-method-based system to create consensus definitions. Vet. Sci..

[2] Pérez-Alenza M.D., Melían C., Ettinger S.J. (2017). Hyperadrenocorticism in dogs. Textbook of Veterinary Internal Medicine.

[3] Zeugswetter F.K., Carranza Valencia A., Glavassevich K., Schwendenwein I. (2021). Patterns of the low-dose dexamethasone suppression test in canine hyperadrenocorticism revisited. Vet. Clin. Pathol..

[4] Bennaim M., Shiel R.E., Forte C., Money C.T. (2018). Evaluation of individual low-dose dexamethasone suppression test patterns in naturally occurring hyperadrenocorticism in dogs. J. Vet. Intern. Med..

[5] Nagata N., Sawamura H., Morishita K., Hosoya K., Yokoyama N., Sasaoka K., Sasaki N., Nakamura K., Ikenaka Y., Takiguchi M. (2022). Urinary corticoid to creatinine ratios using IMMULITE 2000 XPi for diagnosis of canine hypercortisolism. J. Vet. Med. Sci..

[6] Behrend E.N., Kooistra H.S., Nelson R., Reusch C.E., Scott-Moncrieff J.C. (2013). Diagnosis of spontaneous canine hyperadrenocorticism: 2012 ACVIM Consensus Statement (Small Animal). J. Vet. Intern. Med..

[7] Del Baldo F., Corsini A., Tardo A.M., Tirolo A., Sapignoli A., Tumbarello M., Vasylyeva K., Fracassi F. (2024). Hypothalamic-pituitary-adrenal axis recovery after intermediate-acting glucocorticoid treatment in client-owned dogs. J. Vet. Intern. Med..

[8] Galeandro L., Sieber-Ruckstuhl N.S., Riond B., Hartnack S., Hofmann-Lehmann R., Reusch C.E., Boretti F.S. (2014). Urinary corticoid concentrations measured by 5 different immunoassays and gas chromatography-mass spectrometry in healthy dogs and dogs with hypercortisolism at home and in the hospital. J. Vet. Intern. Med..

[9] Schäfer I., Rehbein S., Holtdirk A., Kottmann T., Klein R., Müller E., Thoren-Tolling F. (2023). Diagnostic cut-off values for the urinary corticoid:creatinine ratio for the diagnosis of canine Cushing’s syndrome using an automated chemiluminiscent assay. Vet. Clin. Pathol..

[10] Arenas C., Melían C., Galac S., van den B., Veraa S., Golinelli S., Meij B., Del Magno S., van Nimweger S.A., Zandvliet M., Galac S., Fracassi F. (2024). Cushing’s Syndrome. Canine Endocrinology.

[11] Vaessen M.J., Kooistra H.S., Mol J.A., Rijnberk A. (2004). Urinary corticoid:creatinine ratios in healthy pet dogs after oral low-dose dexamethasone suppression tests. Vet. Rec..

[12] Benchekroun G., de Fornel-Thibaud P., Rodriguez Piñeiro M.I., Rault D., Besso J., Cohen A., Hernandez J., Stamboui F., Gomes E., Garnier F. (2010). Ultrasonography criteria for differentiating ACTH dependency from ACTH independency in 47 dogs with hyperadrenocorticism and equivocal adrenal asymmetry. J. Vet. Intern. Med..

[13] Pollard R.E., Reilly C.M., Uerling M.R., Wood F.D., Feldman E.C. (2010). Cross-sectional imaging characteristics of pituitary adenomas, invasive adenomas and adenocarcinomas in dogs: 33 cases (1988–2006). J. Vet. Intern. Med..

[14] Stolp R., Rijnberk A., Meijer J.C., Croughs R.J. (1983). Urinary corticoids in the diagnosis of canine hyperadrenocorticism. Res. Vet. Sci..

[15] 15 Reimers T.J., Salerno V.J., Lamb S.V. (1996). Validation and application of solid-phase chemiluminescent immunoassays for diagnosis of endocrine diseases in animals. Comp. Haematol. Int..

[16] Singh A.K., Jiang Y., White T., Spassova D. (1997). Validation of nonradioactive chemiluminescent immunoassay methods for the analysis of thyroxine and cortisol in blood samples obtained from dogs, cats, and horses. J. Vet. Diagn. Investig..

[17] Zeugswetter F., Bydzovsky N., Kampner D., Schwendenwein I. (2010). Tailored reference limits for urine corticoid:creatinine ratio in dogs to answer distinct clinical questions. Vet. Rec..

[18] Peterson M.E. (2007). Diagnosis of hyperadrenocorticism in dogs. Clin. Tech. Small Anim. Pract..

[19] Hillebrand J.J., Zhou L., Marcinkus M.A., Datwyler M., Gawel S.H., Martens F., Davis G.J., Heijboer A.C. (2021). Instability of corticotropin during long-term storage—Myth or reality?. Clin. Chem. Lab. Med..

[20] van Vonderen I.K., Kooistra H.S., Rijnberk A. (1998). Influence of veterinary care on the urinary corticoid:creatinine ratio in dogs. J. Vet. Intern. Med..

[21] Nieman L.K. (2015). Cushing’s Syndrome: Update on signs, symptoms and biochemical screening. Eur. J. Endocrinol..

[22] Korchia J., Freeman K.P. (2021). Validation study of canine serum cortisol measurement with the Immulite 2000 Xpi cortisol immunoassay. J. Vet. Diagn Investig..

[23] Kemppainen R.J., Peterson M.E. (1993). Circulating concentration of dexamethasone in healthy dogs, dogs with hyperadrenocorticism, and dogs with nonadrenal Illness during dexamethasone suppression testing. Am. J. Vet. Res..

[24] Russell N.J., Foster S., Clark P., Robertson I.D., Lewis D., Irwin P.J. (2007). Comparison of radioimmunoassay and chemiluminescent assay methods to estimate canine blood cortisol concentrations. Aust. Vet. J..

