# Supplementary material for: Urinary Corticoid-to-Creatinine Ratio 8 Hours After Low-Dose Oral Dexamethasone for the Diagnosis of Cushing’s Syndrome in Dogs
Source: Animals (Basel). 2025 Dec 28;16(1):84. doi: 10.3390/ani16010084 (PMC12784649; doi:10.3390/ani16010084)
Supplement: Supplementary file 1 [file animals-16-00084-s001.zip › S1-Supplementary material O-LDDST.pdf]

Supplementary Material S1
Pituitary-dependent Cushing's syndrome (P-CS)

#	Breed	Sex (F/M)	Age (years)	BW (Kg)	L-AG (mm)	R-AG (mm)	P/B (mm^3)	B-uCC (x10^{-6})	8h-cortisol IV-LDDST	eACTH	ACTHst
1	Poodle	SF	10	5,4	6,7	8,9	0,52	51,5	-	-	+
2	Boxer	F	8	41	8,7	11,2	0,33	93,3	+	-	-
3	Cross-Breed	SF	12	12,5	7,9	12	0,8	63,5	-	-	+
4	Dachshund	NM	8	5,7	5,6	8,6	0,7	285	+	-	-
5	Cross-Breed	NM	9	24,2	8,2	8	0,34	64,2	-	-	+
6	Cross-Breed	SF	12	22,9	7,7	8,9	0,66	20,3	+	-	-
7	Pit Bull	SF	12	24	7,1	5,8	0,4	227	-	-	+
8	Cross-Breed	SF	14	33	9,9	9	0,5	22,7	+	-	-
9	Cross-Breed	SF	12	14,7	7	6,7	0,45	19,9	-	-	+
10	Schnauzer	NM	7	8,4	8,8	5,4	0,66	91,6	+	-	-
11	Poodle	NM	8	7,2	5,6	8	0,21	47,5	-	-	+
12	Pinscher	M	9	4,8	6,5	7,2	0,18	29,9	-	-	+
13	Cross-Breed	SF	7	18,9	6,5	7	0,52	73,4	-	-	+
14	Cross-Breed	SF	11	27	11	8,5	0,66	14,1	+	-	-
15	Cross-Breed	SF	10	30	6,4	8,5	0,44	66,2	+	-	-
16	Poodle	SF	8	5,4	6,6	7,0	1,18	61,7	+	-	-
17	Swiss S.	SF	10	38	9	9,5	0,2	34,4	+	-	-
18	Poodle	NM	14	3,9	6	4,8	-	18,6	-	-	+
19	Poodle	NM	14	4,4	6,7	5,5	-	14	-	-	+
20	Golden	SF	9	39,6	9	4,5	0,28	51,4	+	-	-
21	Maltese	F	6	4,5	5,3	5	0,18	227	+	-	-
22	Boxer	NM	9	33	15	14	0,46	40,3	+	-	-
23	Bichon Fresé	NM	12	9,5	9	6,9	0,64	52,2	-	-	+
24	Cross-Breed	M	11	10	6	6	0,51	36,6	+	-	-
25	Poodle	SF	10	8,7	11,4	10	-	64.6	-	-	+
26	Boxer	SF	11	29	8,8	9,1	-	149	-	-	+
27	Maltes	F	8	5	1	7	-	19,5	+	+	
28	Cross-Breed	M	10	2	4,4	4,3	0,15	35	(-)	-	+
29	Poodle	F	13	5,1	5,7	4,8	0,19	104	+	+	-
30	Pit Bull	SF	11	26,5	8,2	7,9	0,24	46,9	+	+	-
31	Poodle	NM	12	5,1	9	4,5	0,18	17,2	+	+	-
32	Yorkshire T.	F	6	1,3	4,9	4,3	-	145	+	-	-
33	Cross-Breed	SF	9	6,8	4,4	5,3	0,23	19,1	+	+	-
34	Poodle	SF	11	8,6	5,1	4,1	-	25,9	+	+	-
35	Cross-Breed	NM	9	23,8	9,2	9,4	0,2	26,7	+	+	-

36	Poodle	NM	9	3,8	4,9	4,7	-	105	+	-	-
37	Cross-Breed	NM	13	23,6	5	6	-	34,5	+	-	-
38	Poodle	NM	11	8	7	6	-	695	-	-	+
39	Pit Bull	SF	11	9	9,2	11	-	24,4	+	-	-
40	Yorkshire T.	SF	13	4,7	8,9	8	-	320	+	+	-
41	Boxer	SF	11	26,6	9	9,4	-	149	+	-	-
42	Pinscher	SF	13	8	5,5	5,8	-	14,4	-	-	+
43	Boxer	NM	12	33	6,9	9,6	-	41,9	-	-	+
44	Poodle	SF	9	5	10	10	-	424	-	-	+
45	Shetland S.	M	12	10,9	6,6	6,4	-	21,9	-	-	+
46	Dachshund	M	9	8	6	5,8	0,2	57,8	+	-	-
47	Cross-Breed	SF	13	23	10	9	0,32	10,7	+	-	-
48	Yorkshire T.	NM	11	7,3	6,6	7,5	0,28	13,6	-	+	+
49	Shih-tzu	FS	9	7,3	8,6	7,7	0,2	46,7	(-)	-	+
50	Cross-Breed	SF	14	21	11,8	10	0,19	23,2	+	-	-
51	Schnauzer	NM	7	7,9	6,4	7,4	0,21	32,5	-	-	+
52	Poodle	NM	9	4,1	6,3	6	0,18	10,9	-	-	+
53	Cross-Breed	NM	11	23	6,8	6,5	-	46,6	+	-	-
54	Poodle	SF	9	6,3	6,1	6,5	-	10,7	+	-	-
55	Cross-Breed	SF	11	8	6,7	7,0	-	24,9	-	-	+
56	Yorkshire T.	SF	11	6,5	12	9,6	0,15	13,6	+	+	-
57	Beagle	SF	7	16,4	6,6	7,1	0,19	5,3	+	+	-
58	Poodle	NM	12	5,2	5,8	7	-	13,1	-	-	+
59	Poodle	SF	9	8,5	6,7	4,1	-	61,9	-	-	+
60	Fox Terrier	F	10	10	5,4	8,4	0,14	35	+	-	-
61	Poodle	F	7	4,6	6	7	-	90,6	-	-	+
62	Shih-Tzu	SF	11	7,5	6,7	4,5	-	27,7	-	-	+
63	Cross-Breed	NM	9	6	8,8	6,8	-	319	+	-	-
64	Cross-Breed	NM	8	27	9,9	13	0,23	33,4	+	-	-
65	Pinscher	SF	9	7,9	6,0	6,1	0,25	30,9	-	-	+
66	Poodle	NM	10	10	8,6	9,1	-	71,6	+	-	-
67	Schnauzer	SF	8	6,3	7,3	7,1	-	21,9	+	-	-

F: female, **M:** male, **SF:** spayed female, **NM:** neutered male, **BW:** body weight; **L-AG:** left adrenal gland (caudal pole); **R-AG:** right adrenal gland (caudal pole), **P/B:** pituitary-to-brain area ratio; **B-uCC:** baseline urinary cortisol-to-creatinine ratio; **8h-IV-LDDST:** 8-hour cortisol after intravenous low-dose dexamethasone suppression test (> 1.4 μg/dL); **eACTH:** endogenous ACTH (>10 pg/mL); **ACTHst:** ACTH stimulation test (post-ACTH cortisol > 22 μg/dL); **+:** positive for CS; **-:** not performed; **(-):** negative for CS.

Supplementary Material S1
Adrenal-dependent Cushing's syndrome (A-CS)

#	Breed	Sex (F/M)	Age (Years)	BW (Kg)	L-AG (mm)	R-AG (mm)	PB (mm^3)	B-uCC ($x10^{-6}$)	eACTH	8h-cortisol IV-LDDST	Tri/HP (AC)
68	Bichon Fresé	SF	11	9,9	34	5,2	-	220	< 10	+	Tri.
69	Golden	SF	4	45	25	5,9	-	79,1	<10	+	AC
70	Poodle	SF	13	7	17	5,7	-	136	< 10	+	Tri.
71	Cross-Breed	SF	12	9,8	14	4,2	-	9,3	< 10	+	Tri.
72	Poodle	SF	11	8,2	19	3	0,15	12,2	< 10	+	-
73	Cross-Breed	SF	12	9,4	4,8	11	-	9,6	< 10	+	Tri.
74	Poodle	SF	9	5,8	39	4	0,15	37,5	< 10	+	-
75	Poodle	M	14	5,4	6	11,5	-	28,3	< 10	+	Tri.
76	Poodle	SF	9	2,7	34	5	0,16	69,4	-	+	AC
77	Poodle	NM	14	3	9,3	4,8	-	17.3	< 10	+	Tri.
78	Poodle	SF	11	5,7	11,6	3,8	-	11,9	< 10	+	-
79	Jack Russell	NM	12	10	5,4	23*	-	14,1	< 10	+	Tri.
80	Poodle	F	14	5,8	42°	19°	-	258	<10	+	Tri.
81	French Bulldog	SF	12	13	18	4,3	-	42,2	-	+	Tri.
82	English Bulldog	SF	11	26	5,2	19	-	54,1	-	+	AC*
83	Poodle	NM	12	8,1	25	4	-	10,9	<10	+	Tri.
84	Labrador	NF	13	39	47	10•	0,18	88,4	-	+	AC*
85	Beagle	SF	7	24	5,2	10,8	-	7,4	<10	+	Tri.
86	Cross-Breed	SF	14	9,6	12	4,8	-	21,3	< 10	+	Tri.

F: female, **M:** male, **SF:** spayed female, **NM:** neutered male, **BW:** body weight; **L-AG:** left adrenal gland (caudal pole); **R-AG:** right adrenal gland (caudal pole), **P/B:** pituitary-to-brain area ratio; **B-uCC:** baseline urinary cortisol-to-creatinine ratio; **8h-IV-LDDST:** 8-hour cortisol after intravenous low-dose dexamethasone suppression test (> 1.4 µg/dL); **eACTH:** endogenous ACTH (>10 pg/mL); **+:** positive for CS; **-:** not performed; **Tri.:** trilostane treatment; **AC**: adrenocortical carcinoma; **HP:** histopathology diagnosis; *****: invasion of the caudal vena cava (CVC); °: bilateral adrenal tumor; •: resolution of clinical signs and hormonal test results after left adrenalectomy.
