# Supplementary material for: Urinary Corticoid-to-Creatinine Ratio 8 Hours After Low-Dose Oral Dexamethasone for the Diagnosis of Cushing’s Syndrome in Dogs
Source: Animals (Basel). 2025 Dec 28;16(1):84. doi: 10.3390/ani16010084 (PMC12784649; doi:10.3390/ani16010084)
Supplement: Supplementary file 1 [file animals-16-00084-s001.zip › S2-Supplementary Material-O-LDDST.pdf]

Supplementary Material 2

Analytical validation of urinary cortisol measurement by chemiluminescent microparticle immunoassay (CMIA, Architect i2000SR)

Urinary cortisol was quantified using a chemiluminescent microparticle immunoassay (CMIA) on the Architect i2000SR analyzer (Abbott Laboratories, USA).

• **Intra-assay precision (repeatability):** Ten replicates of three canine urine samples with high, medium, and low cortisol concentrations yielded CVs of 2.5%, 4.2%, and 6.0%, respectively, indicating good repeatability.

• **Inter-assay precision (reproducibility):** Four replicates of three urine samples with high, medium, and low cortisol concentrations analyzed on different days yielded CVs of 8.5%, 6.1%, and 28.6%, respectively. Variability was acceptable at medium/high concentrations, while higher CV at low cortisol concentrations reflected expected assay limitations near the lower quantification range.

• **Linearity under dilution:** Serial dilutions of a high cortisol urine sample (1:2 to 1:16) showed excellent proportionality ($r^2 = 0.996$).

Method comparison: Architect i2000SR vs. Immulite 1000 (CLIA, validated for dogs)

A total of 32 paired canine urine samples were analyzed with both assays.

• **Correlation:** Cortisol concentrations measured with the Architect i2000SR and Immulite 1000 were strongly correlated (Pearson r = 0.94, Spearman r = 0.93; $P < 0.0001$) (**Figure 1**).

• **Regression analysis:** The regression equation was Immulite. = 1.06 × Architect + 0.18, indicating minimal proportional bias.

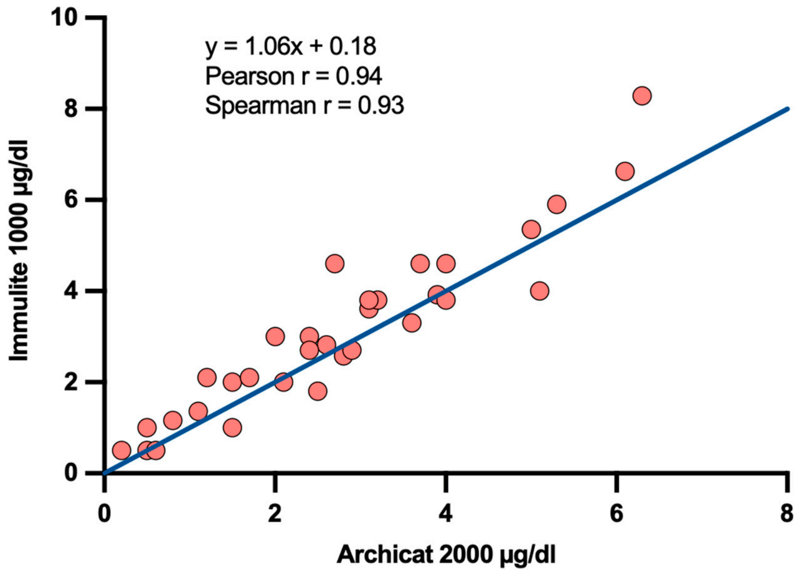

Supplementary Figure S1. Correlation of urinary cortisol concentration measured by Architect i2000SR and Immulite 1000 in 32 paired canine urine samples. Each dot represents one dog. The blue line indicates the linear regression (y = 1.06x + 0.18). Pearson correlation coefficient was r = 0.94 (*P 0.0001*) and Spearman r = 0.93, showing excellent agreement between methods.

Agreement analysis

Bland–Altman analysis demonstrated a mean bias of –0.33 with 95% limits of agreement from –1.58 to 0.91 (**Figure S2**). Data points were evenly distributed around zero without evidence of proportional bias, indicating good agreement between methods.

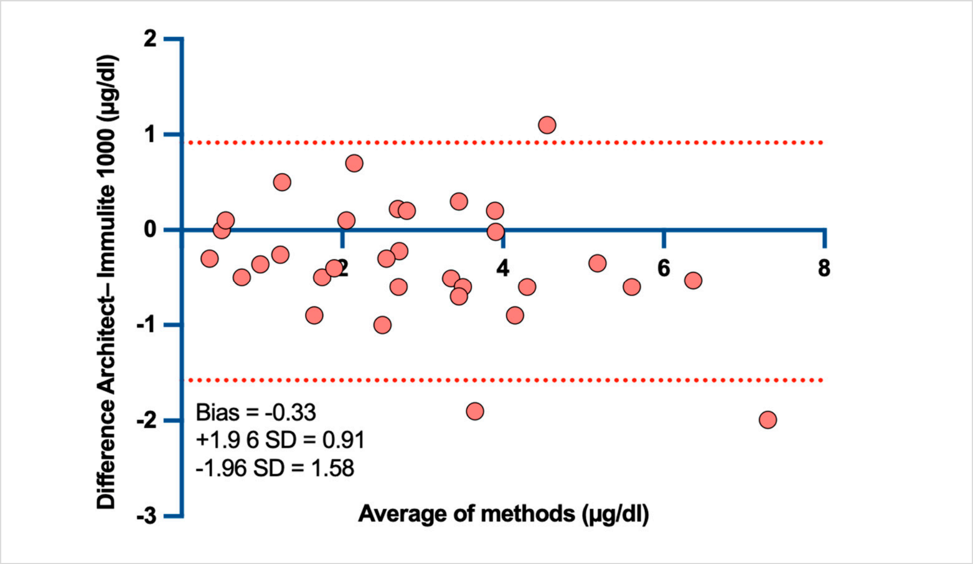

Supplementary Figure S2. Bland–Altman plot comparing Architect i2000SR and Immulite 1000 urinary cortisol measurements. The solid blue line represents the mean bias (–0.33), and dashed red lines represents the 95% limits of agreement (–1.58 to 0.91). Most data points fell within these limits, with no evidence of proportional bias.
